# The Effect of an Alternative Definition of “Percent Highly Annoyed” on the Exposure–Response Relationship: Comparison of Noise Annoyance Responses Measured by ICBEN 5-Point Verbal and 11-Point Numerical Scales

**DOI:** 10.3390/ijerph18126258

**Published:** 2021-06-09

**Authors:** Makoto Morinaga, Thu Lan Nguyen, Shigenori Yokoshima, Koji Shimoyama, Takashi Morihara, Takashi Yano

**Affiliations:** 1 Department of Architecture, Faculty of Engineering, Kanagawa University, Kanagawa 221-8686, Japan; 2 Department of Architectural Design, Interdisciplinary Faculty of Science and Engineering, Shimane University, Shimane 690-8504, Japan; lan@riko.shimane-u.ac.jp; 3 Kanagawa Environmental Research Center, Kanagawa 254-0014, Japan; yokoshima.7c7q@pref.kanagawa.lg.jp; 4 Aviation Environment Research Center, Organization of Airport Facilitation, Tokyo 105-0011, Japan; k-shimoyama@aeif.or.jp; 5 Department of Architecture, National Institute of Technology, Ishikawa College, Ishikawa 929-0392, Japan; morihara@ishikawa-nct.ac.jp; 6 Kumamoto University, Kumamoto 860-8555, Japan; yano@gpo.kumamoto-u.ac.jp

**Keywords:** highly annoyed, ICBEN scales, exposure–response relationship, social survey

## Abstract

Since the development of the 5-point verbal and 11-point numerical scales for measuring noise annoyance by the ICBEN Team 6, these scales have been widely used in socio-acoustic surveys worldwide, and annoyance responses have been easily compared internationally. However, both the top two categories of the 5-point verbal scale and the top three ones of the 11-point numerical scale are correspond to high annoyance, so it is difficult to precisely compare annoyance responses. Therefore, we calculated differences in day–evening–night-weighted sound pressure levels (*L*_den_) by comparing values corresponding to 10% highly annoyed (HA) on *L*_den__%HA curves obtained from measurements in 40 datasets regarding surveys conducted in Japan and Vietnam. The results showed that the *L*_den_ value corresponding to 10% HA using the 5-point verbal scale was approximately 5 dB lower than that of the 11-point numerical scale. Thus, some correction is required to compare annoyance responses measured by the 5-point verbal and the 11-point numerical scales. The results of this study were also compared with those of a survey in Switzerland.

## 1. Introduction

Schultz [1] used the term “percent highly annoyed” (%HA) to define the rate of people who were classified either in the top two categories of a 7-point scale (cutoff value: 71%) or in the top three categories of an 11-point scale (cutoff value: 73%) for measuring noise annoyance. He also emphasized the importance of high annoyance rather than median annoyance, because median annoyance is more influenced by non-acoustical variables than high annoyance. He also pointed out that the median response is much more difficult to translate from one annoyance scale to another and, furthermore, corresponds to no complaint and thus cannot be used for policy purposes.

Schultz [1] showed the synthesized curve relating the day–night-weighted sound pressure level (*L*_dn_) to %HA regardless of the noise source based on social survey data reported at the time. Miedema and Vos [2] proposed separate exposure–response relationships for noise sources through secondary analysis, adding data to the work of Schultz. Then, they defined the upper 28% of annoyance scales (cutoff value: 72%) as %HA, assuming that the scale intervals were equidistant from 0 through 100, regardless of different scale points. Calculation of the 72% cut-off-point was achieved by weighing the annoyance responses of category 4 (very annoyed) with a weight of 0.4. On the other hand, Fields et al. and the Team 6 of the International Commission on Biological Effects of Noise (ICBEN) [3] proposed that 5-point verbal (“not at all,” “slightly,” “moderately,” “very,” and “extremely” annoyed) and 11-point numerical scales (labeling two extremes as “not at all” and “extremely” annoyed) should be used in socio-acoustic surveys, and these scales are adopted by the International Organization for Standardization/technical specifications (ISO/TS) 15666:2003 [4]. Fields et al. [3] proposed defining the top two categories of the 5-point verbal scale (cutoff value: 60%) as high annoyance, because the meanings of “very” and “highly” are similar, but %HA is not defined in ISO/TS 15666:2003.

Initially, Fields et al. constructed the standardized annoyance scales in nine languages in an international joint study [3], and these have since been developed in other languages. Gjestland [5] collected the annoyance scales and question wordings published through 2017 in 17 languages: English, Dutch, French, German, Hungarian, Japanese, Norwegian, Spanish, Turkish, Polish, Danish, Portuguese, Rumanian, Chinese, Korean, Vietnamese, and Thai. Slovenian scales were published in 2018 [6], and to our knowledge, the standardized annoyance scales have been published in these 18 languages only.

Fields et al. [3] measured the intensities of 21 modifiers in English by line marking on a 0–100 scale. Since the average intensity of “Highly” is 79 [3] (p. 661), two categories of the 7-point scale (cutoff value: 71) and three categories of the 11-point scale (cutoff value: 73) corresponding to high annoyance are reasonable considering the range of values indicating %Highly Annoyed. If only the highest category of the 7-point scale (cutoff value, 86) is used to designate a high annoyance, this would result in an extreme response. On the other hand, if the top three categories of the 7-point scale (cutoff value, 57) are included, the response would be classified as median annoyance.

Since the development of the standardized annoyance scales by the ICBEN, they have been used in most socio-acoustic surveys. However, either a 73% cutoff HA (top three categories of the 11-point numerical scale) or a 60% cutoff HA (top two categories of the 5-point verbal scale) has usually been reported in publications. An exception is the study by Wothge et al. [7], who reported the combined effects of aircraft and road traffic noise and of aircraft and railway noise in the Noise-Related Annoyance, Cognition, and Health (NORAH) study. They used only the 5-point verbal scale and demonstrated the relationships between day–evening–night-weighted sound pressure level (*L*_den_) and %HAs for both 60% and 72% cutoffs in their article. The calculation of the 72% cut-off-point was performed according to the method of Miedema and Vos [2]. They investigated differences in *L*_den_ between exposure–response curves for 60% and 72% cutoffs at 10% HA and found that the differences were quite large (8–14 dB). This difference is not negligible, considering that *L*_den_ values in the exposure–response curves at 10% HA were adopted in the World Health Organization (WHO) Environmental Noise Guidelines in 2018 [8].

Guski et al. [9] conducted a systematic review on environmental noise annoyance for the development of the WHO Environmental Noise Guidelines. They selected survey studies through a predefined framework (population, intervention and/or exposure, control, confounder, outcome, and study design; PECCOS) and systematically meta-analyzed them. However, %HAs for 73% and 60% cutoffs were found to coexist in the studies. Of the 15 selected aircraft noise surveys, 14 used a 73% cutoff, and 1 used a 60% cutoff. Of 26 road traffic noise surveys, 23 used a 73% cutoff, and 3 used a 60% cutoff. Of 11 railway noise surveys, the final analysis was performed using data from 10 surveys, of which 4 used a 73% cutoff and 6 used a 60% cutoff. As shown [7], there is a large difference in the *L*_den__%HA relationships between the cutoff values of 60% and 72% as %HA. Therefore, a correction for this difference should be applied to precisely conduct a meta-analysis. While Guski et al. [9] stated that the de facto %HA should be 73%, they analyzed the data shown in the articles without correction, such as for the translation from a 60% cutoff to a 72% cutoff. For aircraft noise, a survey with a 60% cutoff was not used in the final analysis. As for road traffic noise, they showed results excluding surveys with a 60% cutoff as well as Japanese and Vietnamese surveys. However, for railway noise, they showed the representative exposure–response relationships excluding only a shinkansen (bullet train) noise survey in Japan, because the exposure–response relationships cannot be drawn if all the surveys with a 60% cutoff are excluded. Therefore, Guski et al. emphasized the need of a re-evaluation including older data (surveys after 2000 were included in the systematic review).

Brink et al. [10] investigated the following factors to be considered when measuring annoyance: scale type (5-point verbal and 11-point numerical scales), position of the annoyance questions, order of the modifiers of the annoyance scale (ascending or descending), and season (spring or autumn). The value of %HA with a 72% cutoff was obtained according to a method previously described [2]. In terms of scale type, there was lower weighting of the relationship between *L*_dn_ and 72% cutoff HA for responses that fell into the second category from the top of the 5-point verbal scale compared with that between *L*_dn_ and 73% cutoff HA measured by the 11-point numerical scale. Nguyen et al. [11] investigated corresponding relationships between responses measured by 5-point verbal and 11-point numerical scales based on data from 15 social surveys carried out in Japan and Vietnam and compared (1) quadratic regression curves between *L*_den_ and 72% cutoff HA following Miedema and Vos [2], (2) logistic regression curves between *L*_den_ and 73% cutoff HA measured by the 11-point numerical scale, and (3) logistic regression curves between *L*_den_ and 60% cutoff HA measured by the 5-point verbal scale. They showed that curve (1) was consistent with curve (2), which was lower than curve (3).

We have conducted socio-acoustic surveys using both the 5-point verbal and the 11-point numerical scales in Japan and Vietnam since the standardized annoyance scales were proposed by ICBEN. In this paper, we drew three curves by using data from 29 social surveys including the abovementioned 15 surveys that Nguyen et al. [11] used: (a) the exposure–response relationship between *L*_den_ and 60% cutoff HA (top two categories) of the 5-point verbal scale, (b) that between *L*_den_ and 73% cutoff HA (top three categories) of the 11-point numerical scale, and (c) that between *L*_den_ and 72% cutoff HA calculated following Schreckenberg’s method [12], in which responses to the second category from the top of the 5-point verbal scale were randomly divided into two groups, i.e., 40% (HA) and 60% (not HA). Schreckenberg’s method is basically based on the same idea as the method of Miedema and Vos [2], but it is useful when analyzing individual data, such as in logistic regression analysis, which was applied in the present study. Then, differences in *L*_den_ at 10% HA between curve (a) and curves (b) or (c) were calculated. The objectives of this study were to investigate (1) whether some correction is necessary when using a 60% cutoff to obtain the equivalent *L*_den_ at 10% HA of the exposure–response curve using a 73% cutoff and (2) whether there are differences in correction values between 72% and 73% cutoffs, when comparing Japanese and Vietnamese results and with respect to noise sources, if the correction is necessary, and (3) to compare the results with those obtained in Switzerland by Brink et al. [10].

## 2. Method

### 2.1. Dataset

As shown in Table 1, we conducted 29 social surveys over 18 years using the 5-point verbal and 11-point numerical scales proposed by ICBEN. The number of respondents ranged from approximately 200 to 1500, and the response rates ranged from 29% to 99%, which are low for Japan and high for Vietnam. There were 14 surveys conducted in Japan, and 15 in Vietnam. There were five surveys on conventional railway noise, four on shinkansen noise, five on combined noise, nine on aircraft noise, and six on road traffic noise. The conventional railway and shinkansen railway noise surveys and the aircraft and road traffic noise surveys were mainly conducted in Japan and Vietnam, respectively. In the combined noise surveys, we evaluated three kinds of annoyance caused by road traffic, aircraft, and the total (road traffic + aircraft) noises, as well as those caused by conventional railway, shinkansen railway, and the total (conventional + shinkansen railway) noises. Thus, each of the five studies on combined noise surveys was divided into three datasets. In addition, the survey of “2016_KNZ_SR” was divided into two datasets because it was conducted in two different regions. Accordingly, a total of 40 (23 + 3 × 5 + 1 × 2) datasets were reanalyzed. In Japan, respondents were selected using a nearest birthday method on a one person per family basis. On the other hand, in Vietnam, each family member was asked to answer in order of age: father, mother, and other adults over 18 years old. While the distribute–collect and distribute–mail methods were used in Japan, the face-to-face interview method was used in Vietnam.

*L*_den_ was available for all surveys except for the survey of 2001_SAP_CR. In the survey 2001_SAP_CR, only *L*_dn_ was available. Therefore, the difference between *L*_den_ and *L*_dn_ was confirmed using the five railway noise datasets (2002_FUK_CR, 2009_KUM_CR, 2010_KUM_CR, 2011_KUM_CR, 2012_KUM_CB) for which both *L*_den_ and *L*_dn_ were available. As a result, the difference between both metrics was found to be in the range from 0.4 dB to 0.6 dB and is not significant. Therefore, it was judged that the difference between *L*_den_ and *L*_dn_ for conventional railway noise in Japan is small, and *L*_dn_ was used instead of *L*_den_ in the survey 2001_SAP_CR. All noise exposure data were obtained using field noise measurements and distance reduction equations, except for aircraft noise, which was measured at a reference point at each survey site.

### 2.2. Analysis

**Figure 1:**
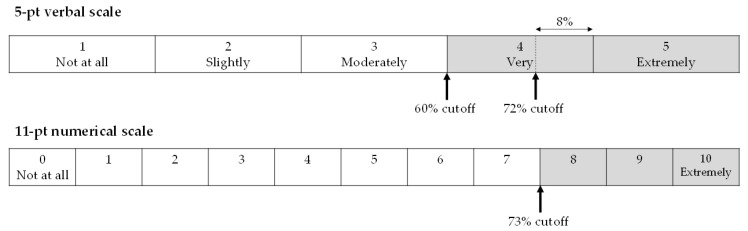
The 5-point verbal and 11-point numerical scales showing 60% and 73% cutoff points for “highly annoyed”.

Figure 1 schematically describes the 5-point verbal and 11-point numerical scales. The top two categories of the 5-point verbal scale and the top three categories of the 11-point numerical scale are defined as Highly Annoyed (HA) in general. If these scales are equidistant, their cutoff points are 60% and 73%, respectively, and the shaded areas show the range of HA. Because the area of a 60% cutoff is larger than that of a 73% cutoff, the HA response to the 5-point verbal scale is easily expected to be larger than that of the 11-point numerical scale.

**Figure 2:**
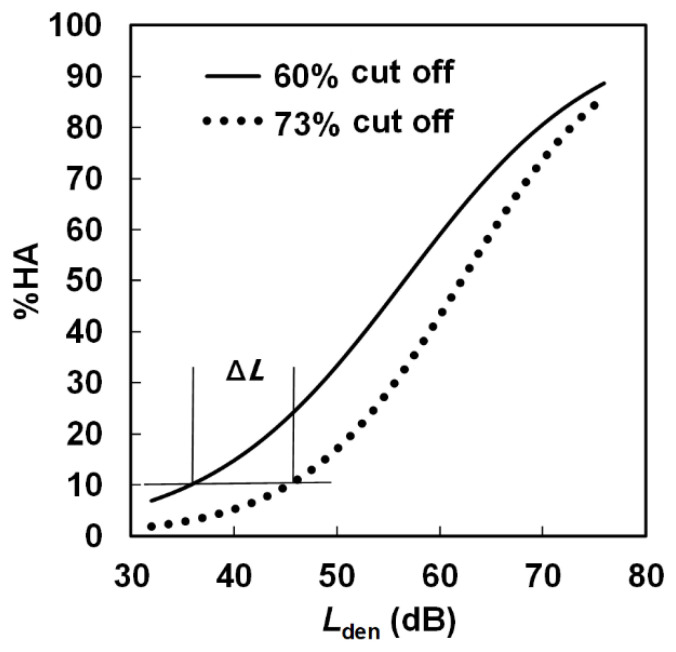
*L*_den_–% highly annoyed relationships for 60% and 73% cutoffs.

The relationships between *L*_den_ and %HA for 60% and 73% cutoffs are schematically shown in Figure 2. WHO guidelines for environmental noises [8] recommend *L*_den_ values corresponding to 10% HA to be the guideline values. Therefore, the *L*_den_ value of a 60% cutoff at 10% HA (abbreviated as *L*_den__10% (60)) is usually smaller than that of a 73% cutoff (*L*_den__10% (73)), and it is expected that there is a difference between the two values (Δ*L* = *L*_den__10% (73) − *L*_den__10% (60)). In this paper, *L*_den_ at 10% HA was estimated from the results of logistic regression analysis with either HA or not HA as the dependent variable and *L*_den_ as an independent variable. In the analysis, *L*_den_ as a continuous variable was applied to individual data above 30 dB of *L*_den_. The difference between 60% and 73% cutoffs is represented by Δ*L*_1_. In addition, following Schreckenberg (2013) [12], responses to the second category from the top of the 5-point verbal scale were randomly divided into two groups: 40% (HA) and 60% (not HA). The averages of the *L*_den_ values in both groups were not significantly different (*t*-test, *p* > 0.05). In this case, the cutoff point was 72%. Logistic regression analysis was applied to the above processed data. The *L*_den_ value of a 72% cutoff at 10% HA is represented by *L*_den__10% (72), and the difference between *L*_den__10% (60) and *L*_den__10% (72) (Δ*L*_2_) was obtained. All analyses were performed using JMP 11 software (SAS Institute Inc., Cary, NC, USA, 2013).

## 3. Results

### 3.1. Analysis of Individual Datasets

We applied logistic regression analysis to the 40 individual datasets and calculated *L*_den__10% (60), *L*_den__10% (73), *L*_den__10% (72), Δ*L*_1_, and Δ*L*_2_. First, we identified the cutoff points for 60% on the 5-point verbal scale, 73% on the 11-point numerical scale, and 72% according to a method reported in the literature [12] and applied logistic regression analysis. Next, *L*_den_ corresponding to 10% HA was estimated from the exposure–response relationships. Finally, we calculated differences between *L*_den__10% (60) and *L*_den__10% (73) and between *L*_den__10% (60) and *L*_den__10% (72) (Δ*L*_1_ and Δ*L*_2_). The results are shown in Table 2. The values of Δ*L*_1_ and Δ*L*_2_ were widespread, particularly when the odds ratio of *L*_den_ was not significant and the area under the curve (AUC) was less than 0.7. For example, 2016_KNZ_SR and 2007_HCM_RT had very small noise exposure ranges from 45 to 55 dB and from 75 to 83 dB, respectively, and thus the slopes of the curves were small. Therefore, the datasets that had odds ratios of *L*_den_ were not significant, and AUCs were less than 0.7 were excluded. The averages of Δ*L*_1_ and Δ*L*_2_ are shown in Table 3. The overall averages of Δ*L*_1_ and Δ*L*_2_ regardless of noise source and country were almost the same at 4.6 dB and 4.3 dB, respectively. While the Δ*L*_1_ and Δ*L*_2_ ranged from 3 to 6 dB depending on noise source, the differences between Δ*L*_1_ and Δ*L*_2_ were small, except for road traffic noise. The averages of Δ*L*_1_ and Δ*L*_2_ in Japan and Vietnam regardless of the noise source were almost the same, while there was a 1.4 dB difference (5.0 dB–3.6 dB) in Δ*L*_2_ between Japan and Vietnam.

### 3.2. Analysis of the Total Dataset

In the results of Table 3, the datasets with low model fit were excluded. In this subsection, multiple logistic regression analysis with HA as the dependent variable and *L*_den_ and dichotomous variables for noise sources as independent variables were applied to the total dataset to confirm whether the same trend for Δ*L*_1_ and Δ*L*_2_ was obtained as in the above subsection. The results of *L*_den__10% (60), *L*_den__10% (73), *L*_den__10% (72), Δ*L*_1_, and Δ*L*_2_ are shown in Table 4. The odds ratios of *L*_den_ were significant (*p* > 0.05), and the AUCs were larger than 0.7 in all three analyses. The values of Δ*L*_1_ and Δ*L*_2_ were slightly larger for road traffic noise than those for the other noises, which were almost the same as those in Table 3. The difference between Δ*L*_1_ and Δ*L*_2_ was around 2 dB at the maximum, and overall, the results were consistent with those in Table 3. The average Δ*L*_1_ and Δ*L*_2_ were 4.8 and 4.9 dBs, respectively.

### 3.3. Difference between Japanese and Vietnamese Data and Swiss Data

**Figure 3:**
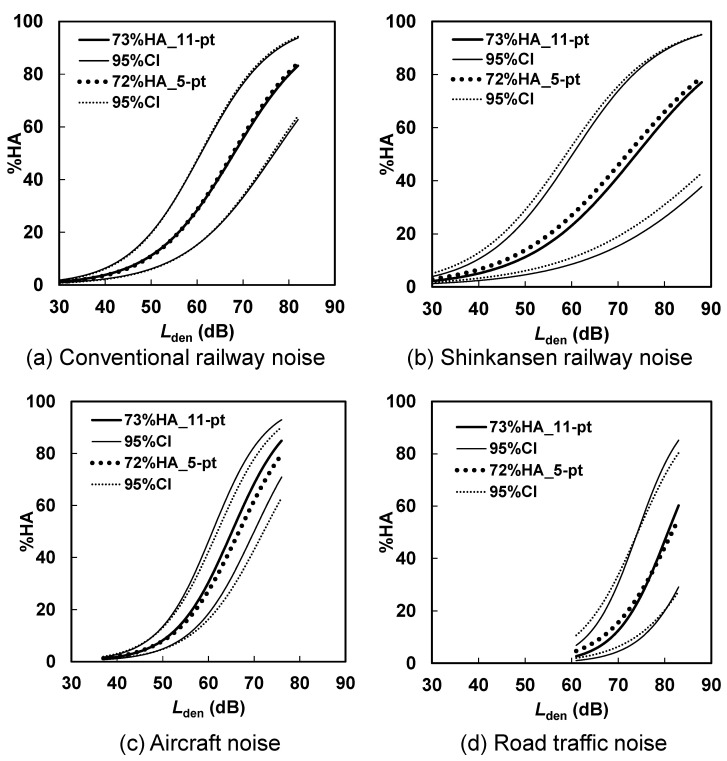
Comparison of exposure–response relationships for environmental noises measured by the 5-point verbal scale and the 11-point numerical scale: (**a**) Conventional railway noise, (**b**) Shinkansen railway noise, (**c**) Aircraft noise, and (**d**) Road traffic noise.

Brink et al. [10] showed that there appeared to be lower weighting of the relationship between *L*_dn_ and 72% cutoff HA calculated from responses of the second category from the top of the 5-point verbal scale compared to that between *L*_dn_ and 73% cutoff HA measured by the 11-point numerical scale, particularly in the range from 62.5 dB to 70.0 dB. The present results are as shown in Figure 3, which compares exposure–response relationships for conventional railway and shinkansen railway noises in Japan and aircraft and road traffic noises in Vietnam between 73% HA measured by the 11-point numerical scale and 72% HA calculated from responses obtained by the 5-point verbal scale. Though the exposure–response relationships for 72% HA by the 5-point verbal scale is slightly higher than that for 73% HA by 11-point scale in Figure 3b and the opposite trend is seen in Figure 3c, there seems to be no consistent difference between the two curves. To investigate in detail the difference between 72% HA using the 5-point verbal scale and 73% HA using the 11-point numerical scale, multiple logistic regression analysis was applied to these data, with HA or not HA as the dependent variable, and *L*_den_, scale type (5-point verbal scale vs. 11-point numerical scale), and the interaction between *L*_den_ and scale type as the independent variables. The results are summarized in Table 5, Table 6, Table 7 and Table 8. While there was no significant difference between the scales in conventional railway noise surveys in Japan (see Table 5) and road traffic noise surveys in Vietnam (see Table 8), there was a significant difference in shinkansen railway noise surveys in Japan (see Table 6) and aircraft noise surveys in Vietnam (see Table 7). Also, only the interaction between *L*_den_ and scale type in the road traffic noise surveys in Vietnam was significant.

Brink et al. [10] converted the 5-point verbal scale and the 11-point numerical scale to an evenly spaced scale ranging from 0 to 100 (discrete point) and examined the correspondence between the two scales. The obtained 5-point verbal scale points were 0, 25, 50, 75, and 100, and the obtained 11-point numerical scale points were 0, 10, 20, 30, 40, 50, 60, 70, 80, 90, and 100. Therefore, we also converted Japanese data into discrete points and compared the range of %HA between those shown in the previous study and those calculated in this study. Here, we analyzed data from Japanese railways and shinkansen noise surveys and Vietnam’s road traffic and aircraft noise surveys, using various datasets. To explain how to convert data to the discrete scale, Table 9 shows an example conversion of a dataset of conventional railway noise conducted in Japan from 2001 to 2017. The values shown in the gray cells in the table indicate the number of respondents for each scale value. The average discrete score of the 5-point verbal scale was calculated considering the weighting of the number of respondents on the corresponding 11-point numerical scale value, and the average discrete score of the 11-point numerical scale was calculated considering the weighting of the number of respondents on the corresponding 5-point verbal scale value. Table 10 shows the average discrete score of the 5-point verbal scale, and Table 11 shows the average discrete score of the 11-point numerical scale of Japanese railways and shinkansen noise surveys and Vietnam’s road traffic and aircraft noise surveys, comparing the results with those of a former study in Switzerland [10]. In Table 10, it was assumed that the range of category 4 is from the midpoint of 4 and 5 to the midpoint of 4 and 3. This range was divided into 40% (HA) and 60% (not HA), and the border of HA and the range of HA were calculated. In Table 11, the range of HA was calculated assuming that the boundary of HA was at the midpoint between categories 7 and 8. As seen in Table 10, the HA range corresponding to the 11-point numerical scale on the 5-point verbal scale (22%) in the Swiss survey was narrower than those in Japanese and Vietnamese surveys (29%–39%), and as seen in Table 11, the HA range corresponding to the 5-point verbal scale on the 11-point numerical scale (33%) in the Swiss survey was wider than those in the Japanese and Vietnamese surveys (23%–28%).

## 4. Discussion

From the results in Table 3 and Table 4, Δ*L*_1_ and Δ*L*_2_ were around 5 dB on average, even though this value was smaller than 8–14 dB in the NORAH study by Wothge et al. [7]. Gjestland criticized the systematic review, particularly for aircraft and road traffic noise guideline values, by Guski et al., who rebutted the criticism [27,28,29,30]. If the guidelines are updated in the future, we hope that these scientific findings will be reflected. While the exposure–response function applied in the WHO guidelines is based on a meta-analysis weighted by the square root of the number of respondents in each dataset [8,9], Gjestland [27] is also critical of the use of weighting in this meta-analysis. As is shown in the introduction, the recommendation value in the WHO guidelines for railway noise is decided using data from 10 surveys of which 4 used a 73% cutoff and 6 used a 60% cutoff. If the correction of 5 dB which can be roughly introduced on the basis of the present study (Table 3 and Table 4) is applied to the six conventional railway noise surveys, which used a 60% cutoff with no weighting of the sample size, the guideline value might be approximately 3 dB larger. Note that this value can be introduced from the simple arithmetic mean of a 5 dB increase over 6 surveys, divided by the total number of surveys, i.e., 10 (5 dB × 6 surveys/10 surveys).

As shown in Table 3 and Table 4, the difference between Δ*L*_1_ and Δ*L*_2_ was found to be small among noise sources and between countries (Japan and Vietnam). This supports findings obtained by Nguyen et al. [11] and indicates the availability of applying Schreckenberg’s method [12] in calculating the 72% cutoff HA from responses evaluated using the 5-point verbal scale.

There was no systematic difference for Δ*L*_1_ and Δ*L*_2_ in regard to the noise source in the results of the analysis using individual datasets, as shown in Figure 3 and Table 5, Table 6, Table 7 and Table 8. This result was also obtained for the average of the total dataset regarding noise sources, shown in Table 3. There was also no large difference for Δ*L*_1_ and Δ*L*_2_ between Japan and Vietnam, as is indicated in Table 3. Accordingly, factors such as noise source and country did not affect the level difference of 5 dB between *L*_den__10% (60) and *L*_den__10% (73) and between *L*_den__10% (60) and *L*_den__10% (72).

Brink et al. [10] indicated a difference between exposure–response curves for 73% HA obtained using the 11-point numerical scale and for 72% HA using the 5-point scale, particularly in the range from 62.5 dB to 70 dB. To investigate the difference in the present datasets in detail, multiple logistic regression analysis was applied to conventional railway, shinkansen railway, aircraft, and road traffic noise survey data separately. Though significant differences were found in exposure–response relationships between 73% HA using the 11-point numerical scale and 72% HA using the 5-point scale in the Shinkansen and aircraft noise surveys, the effect size was small, and the direction was opposite. Therefore, we might consider that there is practically no systematic difference in exposure–response relationships between 72% HA using the 5-point verbal scale and 73% HA using the 11-point numerical scale. As suggested by the results in Table 10 and Table 11, one of the reasons for the difference in the results was the difference in the correspondence between the 5-point verbal and the 11-point numerical scales among the Japanese, Vietnamese, and Swiss surveys. As shown in Table 10, the HA range (22%) in the Swiss survey was narrower than in Japanese and Vietnamese (29–39%) survey, and in Table 11, the HA range (33%) in the Swiss survey was wider than in Japanese and Vietnamese surveys (23–28%). The relatively wider range of HA when using the 11-point numerical scale may be because the *L*_den_–%HA relationship in the 11-point numerical scale was higher than that in the 5-point scale in the Swiss survey. Nonetheless, the difference in exposure–response relationships between 60% HA and 72% or 73% HA is important.

The existence of various definitions of %HA is inconvenient. However, each scale (11-point numerical scale and 5-point verbal scale) has its own merits. For this reason, it is difficult to define %HA choosing only one of the scales. In fact, ISO/TS 15666:2021 [31], which was published in May 2021, points out that we should pay attention to the difference in the definition of %HA by different scales. It is further stated that as an improvement method, the method presented in Reference [2] can be used. It is desirable that such a method of transformation be proposed. The method used in this study is also effective to conduct a secondary analysis based on individual data.

The application of the findings of this study is limited, because the results are based on socio-acoustic surveys conducted only in Japan and Vietnam. Because many surveys using both 5-point verbal and 11-point numerical scales were conducted in developed countries, Δ*L*_1_ and/or Δ*L*_2_ should be validated in those countries.

## 5. Conclusions

In this study, *L*_den_ values at 10% HA were calculated from exposure–response relationships for a 60% cutoff using the 5-point verbal scale, for a 73% cutoff using the 11-point numerical scale, and for a 72% cutoff, weighting responses to the 5-point verbal scale, and the differences in *L*_den_ at 10% HA between 60% cutoff and 73% or 72% cutoff curves were compared. These results were compared with a previous study conducted in Switzerland. This study concludes that:

(1) If 73% or 72% is the de facto standard cutoff point for %HA, the *L*_den_ value at 10% HA for a 60% cutoff should be corrected by adding approximately 5 dB on average in Japan and Vietnam.
(2) There was practically no difference upon correction in regard to noise sources and between Japan and Vietnam.
(3) Though there appeared to be differences in exposure–response relationships between 73% HA using the 11–point scale and 72% HA using the 5-point scale in the Swiss road traffic noise, Japanese Shinkansen noise, and Vietnamese aircraft noise surveys, there was no significant difference in the Japanese conventional railway noise and Vietnamese road traffic noise surveys. We might consider that there is practically no systematic difference in exposure–response relationships between 73% HA determined by the 11-point numerical scale and 72% HA determined by the 5-point verbal scale.

## Figures and Tables

**Table 1:** List of datasets used in the present analysis. Abbreviations in the “Noise source” column indicate the following: CR, conventional railway; SR, shinkansen railway; CB, combined noise source; CA, civil aircraft; RT, road traffic. The numbers in the parentheses in the first column correspond to those of the references at the end of this paper.

Survey ID	Year	Month	Area	Noise Source	Method	Sample Size	Response Rate
2001_SAP_CR [13]	2001	August–October	Sapporo	CR	Distribute-collect	467	69%
2002_FUK_CR [13]	2002	May–June	Fukuoka	CR	Distribute-collect	397	63%
2009_KUM_CR [14]	2009	August–September	Kumamoto	CR	Distribute-collect	206	29%
2010_KUM_CR [14]	2010	July–August	Kumamoto	CR	Distribute-collect	364	29%
2011_KUM_CR [14]	2011	April–May & August–September	Kumamoto	CR	Distribute-collect	704	30%
2003_FUK_SR [15]	2003	Aprili, July	Fukuoka	SR	Distribute-collect	724	66%
2011_KUM_SR [14]	2011	April–May & August–September	Kumamoto	SR	Distribute-collect	735	30%
2013_NAG_SR [16]	2013	July–October	Nagano	SR	Distribute-collect	294	45%
2016_KNZ_SR [17]	2016	November & May	Toyama & Ishikawa	SR	Distribute-collect	1022	52%
2008_HCM_CB [18,19]	2008	August–September	Ho Chi Minh	CB: CA + RT	Interview	682	85%
2009_HAN_CB [18,19]	2009	August–September	Hanoi	CB: CA + RT	Interview	573	76%
2012_KUM_CB [20]	2012	July–August	Kumamoto	CB: CR + SR	Distribute-collect	331	33%
2016_KUM_CB [20]	2016	November–December	Kumamoto	CB: CR + SR	Distribute-collect	399	34%
2017_KUM_CB [20]	2017	July–September	Kumamoto	CB: CR + SR	Distribute-collect	328	26%
2006_KUM_AC [21]	2006	November–October	Kumamoto	CA	Distribute-collect	415	53%
2008_HCM_AC [18,22]	2008	August–September	Ho Chi Minh	CA	Interview	880	87%
2009_HAN_AC [18,22]	2009	August–September	Hanoi	CA	Interview	824	85%
2011_DAN_AC [22]	2011	September	Da Nang	CA	Interview	528	84%
2014_HAN_AC [23,24]	2014	August–September	Hanoi	CA	Interview	891	69%
2015_3_HAN_AC [23,24]	2015	February–Mar	Hanoi	CA	Interview	1121	86%
2015_9_HAN_AC [23,24]	2015	August–September	Hanoi	CA	Interview	1287	99%
2017_HAN_AC [24]	2017	November	Hanoi	CA	Interview	623	96%
2018_HAN_AC [24]	2018	August	Hanoi	CA	Interview	132	88%
2005_HAN_RT [22,25]	2005	August–September	Hanoi	RT	Interview	1503	50%
2007_ISH_RT [26]	2007	November	Ishikawa	RT	Distribute-collect	950	59%
2007_HCM_RT [22,25]	2007	August–September	Ho Chi Minh	RT	Interview	1471	61%
2011_DAN_RT [22]	2011	August–September	Da Nang	RT	Interview	492	82%
2012_HUE_RT [22]	2012	September	Hue	RT	Interview	688	98%
2013_TNG_RT [22]	2013	August–September	Thai Nguyen	RT	Interview	813	81%

**Table 2:** *L*_den_ values at 10% HA for 60%, 73%, and 72% cutoffs of HA, Δ*L*_1_, and Δ*L*_2_. “CB” in the “Survey ID” column indicates the combined noise survey. For example, “CB_CR” indicates data of conventional railway noise in the combined noise survey. The odds ratio for *L*_den_ in each dataset analysis is also shown with the 95% confidential interval (CI). Numbers in red indicate that the AUC values of the logistic regression model are below 0.7.

Survey ID	Noise Range [dB]	A. *L*_den_10%(60)_ [dB]	Odds Ratio (95% CI) [dB]	B. *L*_den_10%(73)_ [dB]	Odds Ratio (95% CI) [dB]	C. *L*_den_10%(72)_ [dB]	Odds Ratio (95% CI) [dB]	Δ*L*_1_ [dB]	Δ*L*_2_ [dB]
2001_SAP_CR	30–80	35.8	1.11 (1.08–1.14)	45.3	1.14 (1.10–1.19)	43.4	1.12 (1.08–1.16)	9.5	7.6
2002_FUK_CR	30–82	41.1	1.10 (1.08–1.13)	50.7	1.11 (1.07–1.14)	46.8	1.09 (1.06–1.12)	9.6	5.7
2009_KUM_CR	30–66	49.2	1.23 (1.14–1.34)	52.8	1.23 (1.13–1.35)	52.1	1.22 (1.13–1.33)	3.6	2.9
2010_KUM_CR	30–69	44.2	1.12 (1.07–1.18)	52.3	1.15 (1.08–1.24)	49.3	1.12 (1.06–1.18)	8.1	5.1
2011_KUM_CR	30–76	45.9	1.14 (1.10–1.20)	48.3	1.13 (1.09–1.18)	50.3	1.15 (1.10–1.21)	2.4	4.4
2012_KUM_CB_CR	31–66	49.1	1.15 (1.09–1.21)	52.3	1.11 (1.06–1.18)	54.2	1.17 (1.10–1.27)	3.2	5.1
2016_KUM_CB_CR	34–63	47.6	1.19 (1.13–1.27)	49.7	1.19 (1.12–1.27)	51.4	1.17 (1.10–1.26)	2.1	3.8
2017_KUM_CB_CR	30–69	41.1	1.06 (1.02–1.10)	48.2	1.08 (1.04–1.13)	52.1	1.05 (1.01–1.10)	7.1	11.0
2003_FUK_SR	36–54	41.8	1.28 (1.21–1.36)	45.6	1.30 (1.22–1.40)	43.9	1.28 (1.20–1.36)	3.8	2.1
2011_KUM_SR	32–70	46.8	1.06 (1.01–1.11)	55.3	1.06 (1.01–1.12)	71.4	1.03 (0.96–1.09)	8.5	24.6
2012_KUM_CB_SR	32–88	55.3	1.07 (1.03–1.10)	58.3	1.07 (1.03–1.11)	64.8	1.08 (1.04–1.14)	3.0	9.5
2013_NAG_SR	46–53	48.5	1.87 (1.52–2.36)	49.2	1.72 (1.36–2.19)	49.4	1.83 (1.44–2.38)	0.7	0.9
2016_KUM_CB_SR	35–63	51.3	1.20 (1.12–1.28)	55.3	1.18 (1.08–1.28)	55.6	1.21 (1.10–1.32)	4.0	4.3
2016_KNZ_SR	45–55	40.5	1.04 (0.94–1.16)	59.0	1.18 (0.94–1.52)	55.5	1.05 (0.92–1.21)	18.5	15.0
2016_TSU_SR	44–55	41.6	1.36(1.21–1.53)	46.6	1.41 (1.23–1.63)	44.2	1.38 (1.22–1.56)	5.0	2.6
2017_KUM_CB_SR	30–64	42.4	1.10 (1.03–1.17)	45.9	1.15 (1.07–1.23)	51.4	1.08 (0.99–1.17)	3.5	9.0
2008_HCM_CB_TO	73–83	72.4	1.47 (1.37–1.58)	74.9	1.58 (1.46–1.71)	75.1	1.38 (1.28–1.49)	2.5	2.7
2009_HAN_CB_TO	70–82	61.1	1.24 (1.18–1.30)	68.2	1.31 (1.25–1.39)	64.4	1.17 (1.13–1.23)	7.1	3.3
2012_KUM_CB_TO	30–88	54.0	1.10 (1.06–1.15)	57.5	1.09 (1.05–1.14)	61.0	1.11 (1.06–1.16)	3.5	7.0
2016_KUM_CB_TO	40–68	54.6	1.18 (1.11–1.26)	58.5	1.20 (1.12–1.30)	58.8	1.19 (1.11–1.28)	3.9	4.2
2017_KUM_CB_TO	34–71	42.8	1.06 (1.02–1.11)	48.7	1.06 (1.02–1.11)	53.4	1.05 (1.00–1.10)	5.9	10.6
2006_KUM_AC	42–55	37.5	1.15 (1.08–1.23)	38.1	1.09 (1.02–1.17)	39.7	1.12 (1.05–1.20)	0.6	2.2
2008_HCM_AC	53–71	56.9	1.32 (1.27–1.39)	60.2	1.28 (1.23–1.35)	59.8	1.20 (1.15–1.25)	3.3	2.9
2008_HCM_CB_AC	53–71	53.9	1.19 (1.15–1.24)	46.6	1.06 (1.03–1.10)	59.9	1.17 (1.13–1.23)	−7.3	6.0
2009_HAN_AC	48–61	39.5	1.11 (1.08–1.15)	49.9	1.18 (1.13–1.23)	47.3	1.11 (1.06–1.16)	10.4	7.8
2009_HAN_CB_AC	48–61	52.9	1.28 (1.20–1.36)	54.8	1.38 (1.28–1.49)	56.4	1.38 (1.27–1.52)	1.9	3.5
2011_DAN_AC	52–64	46.3	1.14 (1.08–1.19)	58.8	1.11 (1.03–1.22)	52.6	1.10 (1.04–1.17)	12.5	6.3
2014_HAN_AC	45–66	49.8	1.25 (1.21–1.30)	53.0	1.20 (1.16–1.25)	52.4	1.20 (1.16–1.25)	3.2	2.6
2015_3_HAN_AC	44–66	47.1	1.22 (1.18–1.26)	48.8	1.18 (1.14–1.21)	49.9	1.19 (1.15–1.22)	1.7	2.8
2015_9_HAN_AC	49–68	50.8	1.38 (1.34–1.43)	51.2	1.28 (1.25–1.32)	51.9	1.25 (1.21–1.28)	0.4	1.1
2017_HAN_AC	38–76	47.7	1.22 (1.17–1.27)	52.2	1.21 (1.17–1.27)	51.4	1.17 (1.14–1.22)	4.5	3.7
2018_HAN_AC	37–71	41.3	1.14 (1.08–1.22)	47.0	1.15 (1.08–1.23)	49.0	1.16 (1.09–1.26)	5.7	7.7
2005_HAN_RT	70–83	62.8	1.28 (1.22–1.34)	62.2	1.14 (1.09–1.19)	58.7	1.11 (1.07–1.15)	-0.6	-4.1
2007_ISH_RT	30–74	49.2	1.10 (1.06–1.13)	55.4	1.07 (1.04–1.11)	55.3	1.10 (1.06–1.14)	6.2	6.1
2007_HCM_RT	75–83	−134.8	1.01 (0.96–1.07)	59.1	1.09 (1.03–1.15)	−97.0	1.01 (0.96–1.06)	193.9	37.8
2008_HCM_CB_RT	73–83	73.2	1.59 (1.48–1.73)	75.6	1.70 (1.56–1.87)	75.2	1.45 (1.34–1.56)	2.4	2.0
2009_HAN_CB_RT	70–82	52.2	1.16 (1.10–1.22)	63.7	1.25 (1.19–1.31)	56.3	1.12 (1.07–1.16)	11.5	4.1
2011_DAN_RT	66–76	69.2	1.50 (1.39–1.63)	73.1	1.52(1.34–1.78)	71.9	1.48 (1.34–1.66)	3.9	2.7
2012_HUE_RT	61–80	64.9	1.15 (1.11–1.20)	74.7	1.15 (1.07–1.24)	70.7	1.14 (1.09–1.21)	9.8	5.8
2013_TNG_RT	61–78	66.3	1.29 (1.23–1.37)	72.1	1.45 (1.32–1.61)	69.7	1.25 (1.17–1.33)	5.8	3.4

**Table 3:** Averages of Δ*L*_1_ and Δ*L*_2_ derived from exposure–response relationships estimated for each dataset.

	Δ*L*_1_ [dB]	Δ*L*_2_ [dB]
All datasets	4.6	4.3
Conventional railway	5.7	5.7
Shinkansen	4.1	3.9
Combined total	4.6	4.3
Civil aircraft	3.4	4.2
Road traffic	5.6	2.9
Japan	4.7	5.0
Vietnam	4.4	3.6

**Table 4:** Averages of Δ*L*_1_ and Δ*L*_2_ derived from exposure–response relationships estimated for each noise source.

	*L*_den_10% (60)_ [dB]	*L*_den_10% (73)_ [dB]	*L*_den_10% (72)_ [dB]	Δ*L*_1_ [dB]	Δ*L*_2_ [dB]
Conventional railway	45.7	49.0	49.6	3.3	3.9
Shinkansen	43.1	46.6	48.9	3.5	5.8
Combined (total)	57.9	63.8	62.1	5.9	4.2
Civil aircraft	45.0	49.1	48.9	4.1	3.9
Road traffic	57.8	65.0	64.5	7.2	6.7
Average				4.8	4.9

**Table 5:** Multiple logistic regression analysis of conventional railway noise surveys from 2001 to 2017 in Japan (AUC = 0.768).

Item	Category	Estimate	Standard Error	*p*-Value	Odds Ratio	95% CI	
						Lower	Upper
Intercept		−7.893	0.277	<0.001			
*L*_den_		0.116	0.005	<0.001	1.123	1.113	1.134
Scale	73% HA_11pt				1.000		
	72% HA_5-pt	−0.023	0.091	0.804	0.978	0.818	1.168
*L*_den_ × Scale		0.003	0.010	0.758			

**Table 6:** Multiple logistic regression analysis of Shinkansen railway noise surveys from 2003 to 2017 in Japan (AUC = 0.669).

Item	Category	Estimate	Standard Error	*p*-Value	Odds Ratio	95% CI	
						Lower	Upper
Intercept		−6.303	0.345	<0.001			
*L*_den_		0.085	0.007	<0.001	1.088	1.074	1.103
Scale	73% HA_11pt				1.000		
	72% HA_5-pt	0.249	0.079	0.002	1.283	1.100	1.498
*L*_den_ × Scale		−0.003	0.014	0.804			

**Table 7:** Multiple logistic regression analysis of aircraft noise surveys from 2008 to 2018 in Vietnam (AUC = 0.724).

Item	Category	Estimate	Standard Error	*p*-Value	Odds Ratio	95% CI	
						Lower	Upper
Intercept		−10.002	0.244	<0.001			
*L*_den_		0.153	0.004	<0.001	1.165	1.156	1.174
Scale	73% HA_11pt				1.000		
	72% HA_5-pt	−0.139	0.043	0.001	0.871	0.801	0.947
*L*_den_ × Scale		−0.013	0.008	0.104			

**Table 8:** Multiple logistic regression analysis of road traffic noise surveys from 2005 to 2013 in Vietnam (AUC = 0.675).

Item	Category	Estimate	Standard Error	*p*-Value	Odds Ratio	95% CI	
						Lower	Upper
Intercept		−13.275	0.424	<0.001			
*L*_den_		0.163	0.005	<0.001	1.178	1.165	1.190
Scale	73% HA_11pt				1.000		
	72% HA_5-pt	0.030	0.043	0.483	1.030	0.948	1.120
*L*_den_ × Scale		−0.036	0.011	0.001			

**Table 9:** Frequency-weighted average of discrete values of the 11-point numerical scale scores on the 5-point verbal scale.

			**11-pt**												
		Discrete	0	10	20	30	40	50	60	70	80	90	100	Sum	Average discrete
	Discrete	Scale value	0	1	2	3	4	5	6	7	8	9	10		
	0	1	436	69	31	14	2	3	0	0	1	0	1	557	3.8
	25	2	171	237	207	174	34	47	7	5	3	0	0	885	18.6
**5-pt**	50	3	31	40	90	186	94	189	75	67	22	4	16	814	41.6
	75	4	4	3	7	20	13	71	45	105	112	23	28	431	66.6
	100	5	3	1	2	2	1	8	3	20	54	35	185	314	89.5
	Sum		645	350	337	396	144	318	130	197	192	62	230		
	Average discrete		10.0	23.6	30.9	38.8	46.0	52.7	58.5	67.8	78.0	87.5	93.0		

**Table 10:** Frequency-weighted average of discrete values of the 11-point numerical scale scores on the 5-point verbal scale.

	5-Point Scale Value					Range of Category 4	Border of Highly Annoyed	Range of Highly Annoyed
	1	2	3	4	5			
	The Number of Respondents							
Road traffic 2012–2013, Switzerland	6	25	52	79	95	22%	78%	22%
Conventional railway 2001–2017, Japan	4	19	42	67	89	24%	69%	31%
Shinkansen 2003–2017, Japan	4	16	34	60	81	24%	61%	39%
Road traffic 2005–2013, Vietnam	23	41	50	70	83	17%	70%	30%
Civil aircraft 2008–2018, Vietnam	12	34	43	72	89	23%	71%	29%

**Table 11:** Frequency-weighted average of discrete values of the 5-point verbal scale score on the 11-point numerical scale.

Survey ID	11-Point Numerical Scale Value											Border of Highly Annoyed	Range of Highly Annoyed
	0	1	2	3	4	5	6	7	8	9	10		
	The Number of Respondents												
Road traffic 2012–2013, Switzerland	2	13	22	28	39	48	52	63	71	77	86	67%	33%
Conventional railway 2001–2017, Japan	10	24	31	39	46	53	58	68	78	88	93	73%	27%
Shinkansen 2003–2017, Japan	11	27	33	40	49	56	62	72	81	87	95	77%	23%
Road traffic 2005–2013, Vietnam	14	24	26	36	44	53	63	71	77	82	87	74%	26%
Civil aircraft 2008–2018, Vietnam	9	22	27	33	40	49	57	68	75	82	91	72%	28%

## Data Availability

The data for this secondary analysis were provided by the Socio-Acoustic Survey Data Archive (SASDA), Institute of Noise Control Engineering/Japan. “http://www.ince-j.or.jp/old/04/04_page/04_doc/bunkakai/shachodata/?page_id=972”.
